# Electrolytic Corrosion Behavior of 20 Cu-20 Ni-54 NiFe_2_O_4_-6 NiO Cermet with Interpenetrating Structure at 880 °C and 960 °C

**DOI:** 10.3390/ma15155377

**Published:** 2022-08-04

**Authors:** Yu-Qiang Tao, Zhi-You Li, Hui-Wen Xiong, Min-Jie He, Bing-Xin Wang

**Affiliations:** 1 School of Chemistry and Chemical Engineering, University of South China, Hengyang 421001, China; 2 Hunan Key Laboratory for the Design and Application of Actinide Complexes, Hengyang 421001, China; 3 State Key Laboratory of Powder Metallurgy, Central South University, Changsha 410083, China

**Keywords:** metal matrix composites, erosion, anodic dissolution, inert anode, Al electrolysis

## Abstract

Here, 20 Cu-20 Ni-54 NiFe_2_O_4_-6 NiO (wt%) cermets were prepared via the powder metallurgy process, and the electrolytic corrosion behavior of the cermets at 880 °C and 960 °C was studied through the microstructure analysis by SEM and EDS. Results show that the ceramic phase is seriously affected by chemical corrosion at 880 °C electrolysis, and it is difficult to form a dense ceramic surface layer. A dense ceramic surface layer is formed on the bottom of the anode electrolyzed at 960 °C, and the dense layer thickens with the extension of the electrolysis time. The formation of the dense surface layer is mainly caused by the oxidation of Ni. The oxidation rate of the metallic phase and the corrosion rate of the ceramic phase have an important effect on the formation of the dense layer. In the corrosion process of NiFe_2_O_4_ phase, preferential corrosion of Fe element occurs first, and then NiO phase is precipitated from NiFe_2_O_4_ phase. After the NiO is dissolved and corroded, the NiFe_2_O_4_ grains collapse and the ceramic phase peels off from the anode.

## 1. Introduction

At present, aluminum electrolysis is mainly carried out in the Hall–Héroult process, in which the electrolysis temperature is about 960 °C, and the anode is mainly composed of carbon. During the electrolysis process, the carbon anode is consumed, as shown in Formula (1) [1]. About 1.5 tons CO_2_ is generated to produce one ton Al for the anode consumption [2]. If the carbon anode is replaced by an inert anode which is not consumed during electrolysis, the CO_2_ emission in the aluminum product process would be significantly reduced. Meantime, a lot of oxygen is produced, as shown in Formula (2). Combined with the clean energy sources such as hydropower as the electricity, green production of aluminum electrolysis can be realized.

2 Al_2_O_3 (non-aq)_ + 3 C _(s)_ = 4 Al _(l)_ + 3 CO_2 (g)_ (1)

2 Al_2_O_3 (non-aq)_ = 4 Al _(l)_ + 3 O_2 (g)_ (2)

The main candidate materials for inert anode are NiFe_2_O_4_-based cermet, Cu alloy, and Ni alloy [3,4,5,6,7,8,9]. On 10 May 2018, Alcoa and Rio Tinto announced world’s first carbon-free aluminum smelting process, and planned for sale beginning in 2024 [10]. A pilot plant for industrial testing with the use of inert anode had been established by RUSAL, and over 1000 tons Al had been produced [11]. On 10 June 2020, RUSAL declared they began testing a new generation of 140 KA inert anode aluminum electrolysis cell [12]. However, no more detail technique has been reported until now.

Poor thermal shock resistance and low electrical conductivity are the main problems for the ceramic-based cermet anode, which were found during the pilot plant test [13]. Increasing the metallic phase content (more than 25 wt%) is an effective measure to solve those problems. It was confirmed that Ni-Fe-Al_2_O_3_ cermet with a metallic phase content of 65 wt% had sufficient machining performance, good thermal shock resistance, and was easily connected to the power supply bar [14]. The electrical conductivity of Cu-NiFe_2_O_4_-Cu_2_O-NiO cermet with a metallic phase content of about 34 wt% was up to 1503 S/cm, which is much higher than that of the carbon anode (200 S/cm) [7]. Cu-Ni-NiFe_2_O_4_ cermet with high metallic phase content also had good electrical conductivity and mechanical properties. It kept intact when dropped from 1.5 m height [15,16].

The oxidation resistance and molten salt corrosion resistance of ceramic phase are generally better than that of the metallic phase. The metallic phase is easily oxidized at high temperature in air and is prone to electrochemical corrosion. As the metallic phase content increases, it would face another problem that of corrosion resistance. Some studies about the oxidation and corrosion behaviors of the cermets with high metallic phase content were reported. The influence of oxidation temperature on the oxidation behavior of 8 Ni-32 Cu-54 NiFe_2_O_4_-6 NiO cermet was studied, and the results revealed that the Cu_2_O content in the outermost layer and the oxidation depth increased with increasing the oxidation temperature [17]. The 20 Ni-20 Cu-54 NiFe_2_O_4_-6 NiO cermet had a good oxidation resistance in air at 880 °C, and the oxidation scale was continuous and dense [18]. The electrolytic corrosion performance of Ni-Fe-Al_2_O_3_ anode with a high metallic phase content was studied, and the annual corrosion rate of the anode was about 1.87 cm [14]. Electrolytic corrosion of 40 Ni-30 Fe-30 WC cermet used as a coating on stainless steel was evaluated at 850 °C, and the annual corrosion rate was 5.8 cm [19], higher than the requirement of inert anode (1 cm/y). NiFe_2_O_4_-M cermets with good thermal shock resistance were also evaluated briefly, and an Al product could be obtained with a purity of 97.48%. However, the content of Fe impurity in Al was up to 1.33%, higher than the requirement of Al production [16]. The influence of Fe content on the corrosion resistance of Cu-Ni-NiFe_2_O_4_-NiO cermet anode was studied under low temperature electrolysis [20,21]. A respectable amount of Fe content was conducive to the formation of a dense ceramic surface, which conduce to the anode self-healing, indicating that the corrosion resistance was greatly affected by the anode composition. Until now, the electrolysis temperature of the cermet anode with high metallic phase content has usually been below 900 °C. There have been few studies on the electrolytic corrosion behavior of the cermet with a high metallic phase content under both high and low temperature conditions.

In our previous studies [17], a dense 32 Cu-8 Ni-54 NiFe_2_O_4_-6 NiO cermet could be prepared by a powder metallurgy process at an appropriate sintering temperature, but the out-diffusion of copper was serious in air at high temperature, especially above 950 °C. Moreover, increasing the Ni content in the metallic phase could improve its oxidation resistance. The 20 Cu-20 Ni-54 NiFe_2_O_4_-6 NiO cermet without Fe addition had good oxidation resistance in air at 880 °C, and the oxide layer thickness of the cermet and oxidation time presented a parabolic relationship [18]. The corrosion resistance of 20 Cu-20 Ni-54 NiFe_2_O_4_-6 NiO cermet at 880 °C and 960 °C has not yet been studied. In order to further explore the corrosion behaviors under different temperatures of the cermet anode with a high metallic phase content, the 20 Cu-20 Ni-54 NiFe_2_O_4_-6 NiO cermet was prepared based on a previous study, and its electrolytic corrosion behaviors at 880 °C and 960 °C were studied through the microstructure analysis in this paper.

## 2. Experimental Procedures

### 2.1. Preparation of the Cermets

Briefly, 20 Cu-20 Ni-54 NiFe_2_O_4_-6 NiO cermets were prepared by powder metallurgy, as described in the previous report [17]. NiFe_2_O_4_-10NiO ceramic composed of 90 wt% NiFe_2_O_4_ and 10 wt% NiO was synthesized by calcining in air at 1200 °C from commercial Fe_2_O_3_ (99.60%, 0.75 μm, JFE Chemical Co., Tokyo, Japan) and NiO (77.64 wt% Ni, Jinchuan Group Ltd., Jinchang, China) powders. Cu-50 Ni powders of composition 50% Cu/50% Ni (wt%) were prepared by chemically coating Ni on Cu powder (99.5%, 9.86 μm, Gripm Advance Materials Co., Beijing, China). The ceramic and metal powders were then mixed with a mass ratio of 6 to 4 and ball milled for 4 h in water and zirconia media containing 1.2 wt% poly (vinyl alcohol) (PVA). The dried powders were pressed into cylindrical blocks using cold isostatic pressing at 150 MPa. Sintering was performed at 1200 °C for 4 h in N_2_ atmosphere with residual oxygen partial pressure of ~10 Pa using a heating rate of 1.4 °C/min and cooling rate of 1 °C/min.

### 2.2. Pre-Oxidation Treatment and Electrolysis Tests

The as-sintered cermets were polished using 1500# abrasive paper before pre-oxidation. Then, the cermets were oxidized in a muffle furnace in air at 880 °C for 0, 4, 24, 48 h, respectively, donated as PO-0, PO-4, PO-24, PO-48.

**Figure 1:**
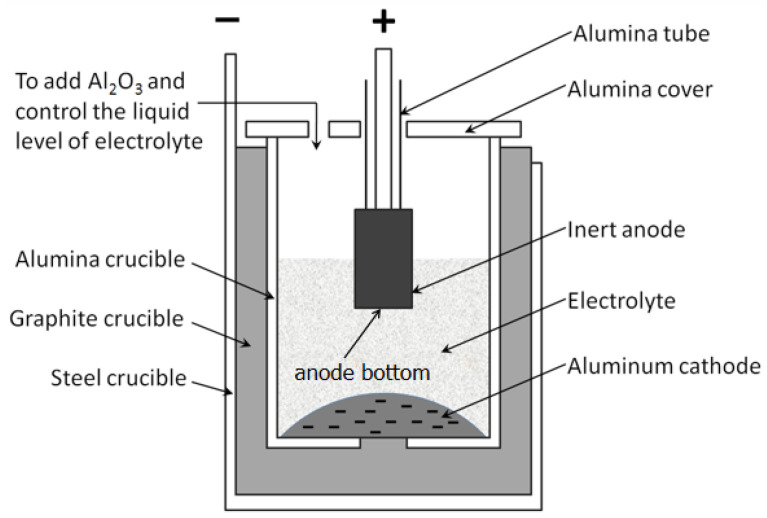
Schematic structure of the electrolysis cell.

The process of electrolysis test was similar to the previous report [22]. The pre-oxidized anode was bonded to the stainless-steel guide bar. Subsequently, the assembled anode was heated together with the electrolyte, and the anode was 10 mm above the electrolyte in the heating process, as shown in Figure 1. After the electrolyte was heated to the electrolysis temperature for 0.5 h, the anode was immersed in the electrolyte for about 20 mm. The electrolysis temperature was 880 °C and 960 °C, respectively, and the corresponding electrolyte composition and parameter are shown in Table 1. The anodes electrolyzed at 880 °C and 960 °C were respectively marked as LT-x-y and HT-x-y, in which x was the pre-oxidation time and y was the electrolysis time, as shown in Table 2. After y hours electrolysis, the anode was raised over the electrolyte, then the electrolysis cell was cooled, and the electrolysis test was terminated. The current density was 1.0 A/cm^2^, based on the bottom area of the anode. The cell voltage was recorded utilizing multimeter with storage function. After electrolysis, the anodes were cut open along the axial direction and polished for further analysis.

### 2.3. Characterization

The phase compositions were identified by X-ray diffraction (XRD) (Rigaku D/max 2550VB+, Rigaku Corporation, Tokyo, Japan) with Cu K_α_ radiation (λ = 0.154 nm). Following the polishing, the cross-sectional microstructures were examined by scanning electron microscopy (SEM) (FEI Quanta 200, FEI Company, Eindhoven, The Netherlands), and the element contents in different phases were analyzed by energy dispersive spectroscopy (EDS) (EDX-GENESIS 60S, EDAX Company of America, Mahwah, NJ, USA). The corrosion depths were measured through the SEM images.

## 3. Results and Discussion

### 3.1. Microstructures of the As-Sintered Cermets

**Figure 2:**
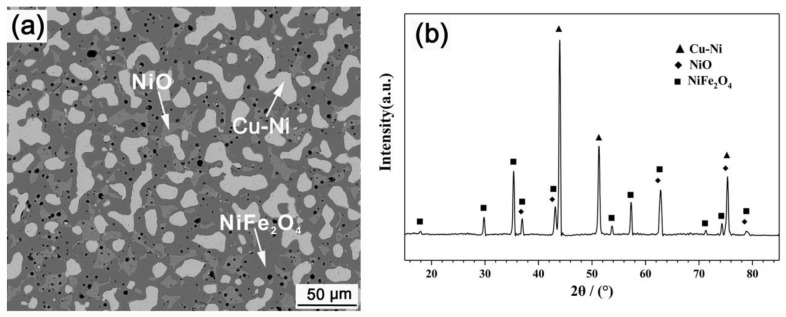
Micrograph (**a**) and XRD pattern (**b**) of the as-sintered cermet.

Figure 2 shows the micrograph and XRD pattern of the as-sintered cermet. The sample is predominantly composed of NiFe_2_O_4_, NiO and Cu-Ni phases. Little pores exist. Most pores are spherical, and the pore size is less than 5 μm. Part of the metallic grains are worm-like. EDS shows that the Ni/Cu atomic ratio in the metallic phase is about 1.12, and the metallic phase contains 2.86 at% Fe. The NiO phase is irregular, in which the Fe/Ni atomic ratio is 0.14. The Fe/Ni atomic ratio in NiFe_2_O_4_ phase is 2.89, which is higher than the theoretical value of 2. 

### 3.2. Microstructures of the Pre-Oxidation Cermets

**Figure 3:**
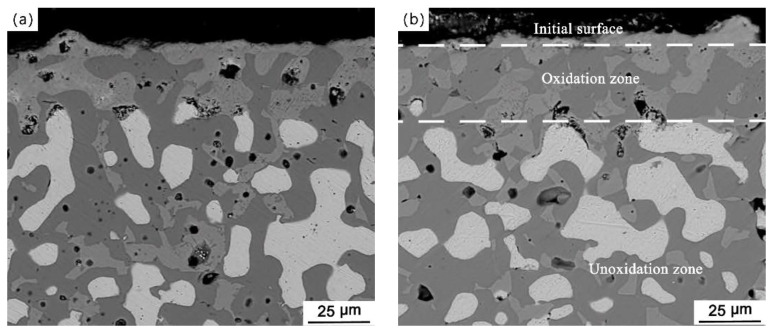

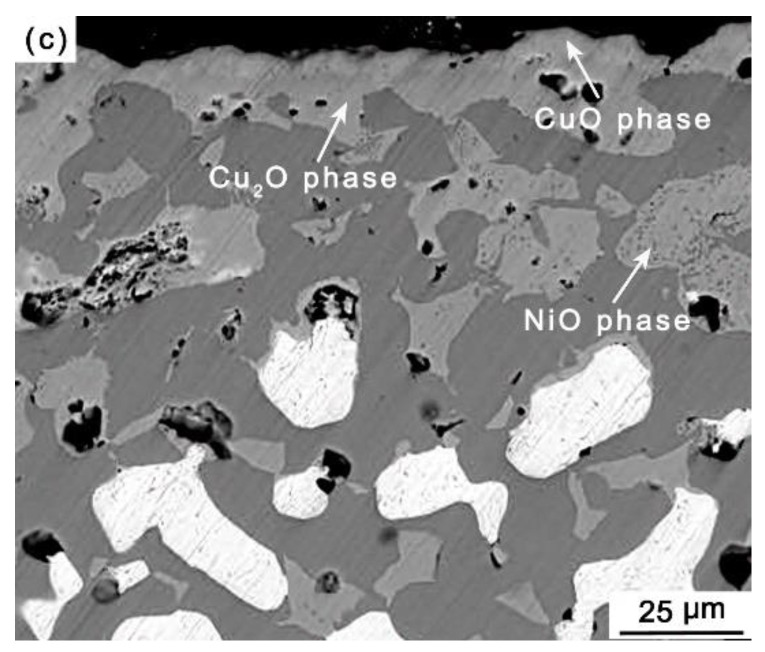
Micrographs of the pre-oxidized cermets, (**a**) PO-4, (**b**) PO-24, (**c**) PO-48.

Figure 3 shows the cross-sectional microstructures of PO-4, PO-24, and PO-48. An oxide scale is formed outside the original surface. There is no metallic phase near the original surface, indicating that both external oxidation and internal oxidation are occurred. For PO-4, only the metallic particles exposed to air are oxidized, and the internal oxidation depth is about 30 μm. The internal oxidation depths of PO-24 and PO-48 are similar, i.e., approximately 45 μm, indicating that the sintered cermet has good oxidation resistance in air. The pores in the internal oxidation zone are irregular and slightly larger than those in the non-oxidized zone, which is caused by the external oxidation of the metallic phase. The oxide scale is relatively dense, and no connecting pore is found, resulting in the good oxidation resistance.

### 3.3. Electrolytic Corrosion Behavior at 880 °C

**Figure 4:**
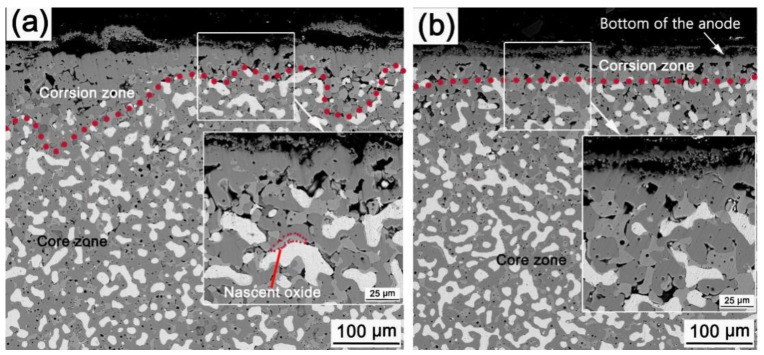
Micrographs of LT-0-15 (**a**) and LT-4-15 (**b**). Red line is the boundary line between the corrosion zone and core zone.

Figure 4 shows the microstructures of LT-0-15 and LT-4-15. Some pores with a size of about 20 μm exist in the corrosion zone for both anodes, and they are are larger than that of the core zone (uncorroded zone). The EDS results show that the electrolyte constituent elements F, Al, Ca are found in the pores. Few metallic grains could be found in the corrosion zone. The thickness of the corrosion layer of LT-4-15 is thinner than that of LT-0-15. The outermost oxide layer formed in the pre-oxidation process has been completely corroded. Compared with LT-0-15, the corrosion of the ceramic phase of LT-4-15 is more uniform, and the thickness of the corrosion zone is more even. For LT-0-15, the spinel phase next to electrolyte is fragmented but still connected to the anode body. A bubbling phenomenon exists in the outermost corrosion layer. A thin oxide layer named nascent oxide is found on the surface of some metallic grains, as shown in the inset of Figure 4a. Some metallic grains directly connect with the large pores in the corrosion zone, in which the Ni/Cu atomic ratio is about 0.58, just half of that located in the core zone. This indicates that Ni is preferentially corroded, which is consistent with the results reported by Cassayre et al. [23].

**Figure 5:**
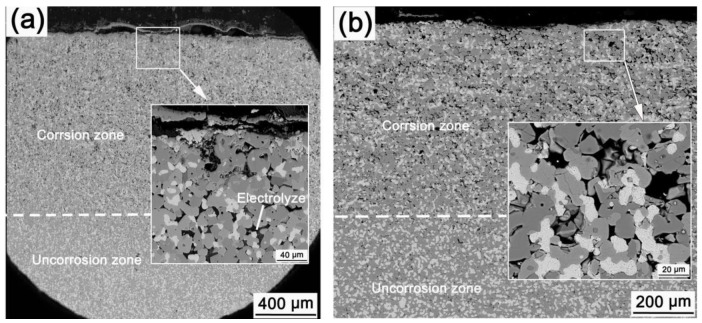
Micrographs of LT-4-25 (**a**) and LT-24-25 (**b**).

Figure 5 shows the microstructures of LT-4-25 and LT-24-25. Corrosion depths of LT-4-25 and LT-24-25 are 1320 μm and 680 μm, respectively. The morphologies of the corrosion zone of the two anodes are similar, and no dense ceramic layer is found on the anode surface. The corrosion zone is porous, and electrolyte is found in the pores. A large amount of metallic phase is still existed in the corrosion zone, and some metallic particles are directly connected to the pores or electrolyte. There is no strip-shaped metallic oxide on the surface of the metallic phase. This suggests that the oxidation rate of the metallic phase is slower than the corrosion rate. According to EDS, the atomic ratios of Ni/Cu in the metallic phase that located in the corrosion zone of LT-4-25 and LT-24-25 are 0.34 and 0.25, respectively, which are about half that of LT-4-15. This further confirms that Ni in the metallic phase is preferentially corroded.

Comparing Figure 4b and Figure 5a, the corrosion depth of LT-4-25 is much larger than that of LT-4-15. In the initial stage of electrolysis, the corrosion of the anode is relatively slight. As the electrolysis proceeds, the pre-oxidation layer is gradually corroded. After a certain period of electrolysis, the oxide scale is completely corroded, because the corrosion rate is larger than the formation rate of the oxide scale. Then, electrochemical corrosion of the metallic phase occurs, and the electrolyte penetration channel is formed, which aggravates the anode corrosion. Finally, the corrosion of the anode becomes more and more acute with the extension of the electrolysis time. This shows that a proper extension of the pre-oxidation time is useful to delay the rapid electrochemical corrosion of the anode. However, the pre-oxidation treatment is not a long-term solution because of the superior oxidation resistance of the cermet.

It is hard to form a dense ceramic layer on the surface when electrolyzes at 880 °C from the above results. This is due to the corrosion rate of the ceramic phase being greater than the growth rate of the oxide scale. The thicknesses of the oxide layers of PO-4 and PO-24 are approximately 30 μm and 45 μm, respectively, as shown in Figure 3. The outermost layer of the oxide scale is composed of CuO and the internal oxidation layer is composed of Cu_2_O, NiO, and NiFe_2_O_4_. The newly formed NiO fails to form a continuous compact layer with the added ceramic phase in the oxidation zone. Further, Cu was continuously oxidized. The corrosion resistance of CuO and Cu_2_O is worse than that of NiFe_2_O_4_ and NiO [24,25]. CuO and Cu_2_O would preferentially be dissolved corrosion and then the electrolyte penetration channels are formed. The corrosion of ceramic phase would be aggravated in a high AlF_3_ concentration electrolyte, as shown in formulation 3 [26], leading to a much higher dissolution rate of the oxide scale than the oxide scale growth rate. As the electrolysis proceeds, the pre-oxidized scale is gradually dissolved and loses its protective effect. As shown in Figure 4, the oxide scale formed by 4 h pre-oxidation has been corroded after electrolysis for 15 h, and the oxide formed through internal oxidation has also begun dissolving, forming a porous surface layer. The ceramic phase around the metallic phase is dissolved and corroded with extension of the electrolysis time, and the metallic phase directly contacts with the electrolyte and starts electrochemical corrosion. As the electrolysis time prolongs, the corrosion depth increases. Prolonging the pre-oxidation time will make the oxide scale thickening, and it would take longer for the oxide scale to be dissolved and corroded. However, the oxide scale eventually is corroded, and the metallic phase directly contacts with the electrolyte and occurs electrochemical corrosion after a specified period of electrolysis. The electrochemical corrosion of the metallic phase is much faster than the dissolution corrosion of the oxides, so the corrosion depth of the anodes with a short pre-oxidation time and long electrolysis time is very serious.

6NiO·Fe_2_O_3_ + 4AlF_3_ = 6NiF_2_ + 6Fe_2_O_3_ + 4Al + 3O_2_ (3)

### 3.4. Electrolytic Corrosion Behavior at 960 °C

**Figure 6:**
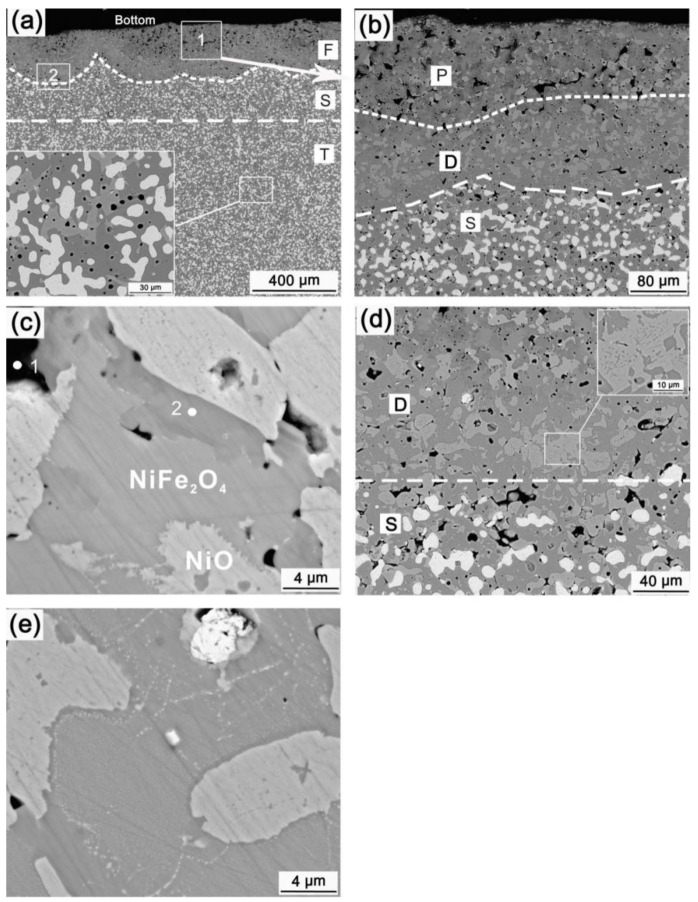
(**a**) Micrographs of HT-48-15, (**b**–**e**) are the magnifications of selection zone 1 in (**a**), zone P in (**b**), selection zone 2 in (**a**), zone D in (**b**), respectively.

**Figure 7:**
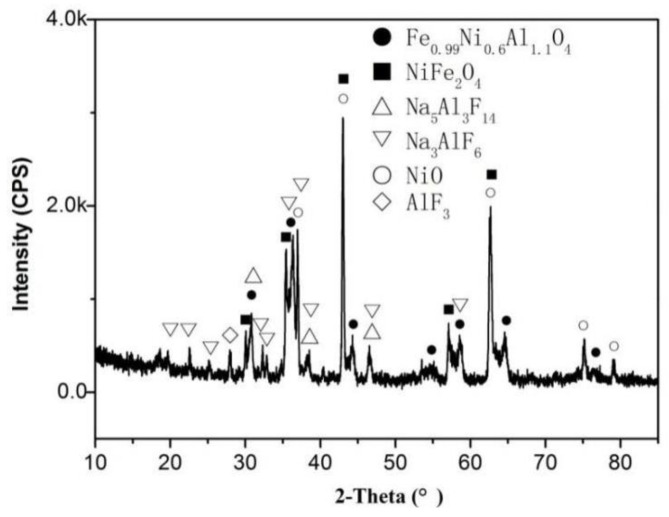
XRD pattern of the bottom surface of HT-48-15.

Figure 6 shows the bottom microstructure of HT-48-15. The anode is composed of three zones F, S and T with different microstructures. No metallic phase is found in zone F, whose thickness is uneven. Zone F is composed of two parts, P and D, as shown in Figure 6b. There are some large pores in zone P, and EDS shows that the pores (point 1 in Figure 6c) contain electrolyte constituent elements such as F, Al and Na. A phase with high contrast (point 2 in Figure 6c) is found in zone P, which is mainly distributed around NiO phase and the pores. EDS shows that this phase is mainly composed of O, Fe, Ni, Al elements and a small amount of Cu element (about 1.13 wt%). Figure 7 is the XRD pattern of the anode bottom surface. The anode bottom surface is mainly composed of NiFe_2_O_4_, NiO, and Fe_0.99_Ni_0.6_Al_1.1_O_4_ except for the electrolyte. Combined with XRD, the dark color phase is the solid solution Fe_0.99_Ni_0.6_Al_1.1_O_4_ of NiAl_2_O_4_ and FeAl_2_O_4_, which was also reported in previous work [22]. The edges of part NiO grains are jagged, as shown in Figure 6c, which confirms that NiFe_2_O_4_ grain could swallow the adjacent NiO grain during electrolysis process [2]. White fine granular substances are also found at the boundary of NiFe_2_O_4_ grains. Zone D is relatively dense, with a thickness of approximately 100 μm, which is much thicker than that of the oxide scale formed in the 48 h pre-oxidation process. It is mainly composed of NiO and NiFe_2_O_4_ phases. A small amount of dark gray aluminate phase can also be found near zone P. Compared with zones P and S, zone D has more NiO and larger particle size. Some NiO grains contain obvious NiFe_2_O_4_ precipitates. In zone D, there are many small particles with contrast close to NiO phase in NiFe_2_O_4_ grains and on the grain boundaries, as shown in Figure 6e, which has not been reported previously. EDS shows that the NiFe_2_O_4_ grains containing precipitates are also composed of four elements Ni, Fe, O, and Cu. The atomic ratio of Ni:Fe:O:Cu is 12.69:25.75:61.01:0.56. NiO in zone S is greater and coarser than that of zone T. The Ni/Cu mass ratio of the metallic phase in zone S is 0.53, which is about half of that in the zone T (1.07). Some pores are around the metallic grains, and no electrolyte constituent elements are found in the pores. These pores should be formed by outward diffusion oxidation of Cu element [17]. Zone T has the exact same morphology as the core and is an uncorroded zone.

Table 3 shows the Fe/Ni atomic ratios of NiFe_2_O_4_ and NiO phases in different zones in Figure 6. From the surface to the core, the Fe/Ni ratio in NiFe_2_O_4_ phase decreases gradually. The Fe content of NiO phase in zone P is less than that of other zones. The decrease of Fe element contents of both NiFe_2_O_4_ and NiO phases in the corrosion zone indicates that Fe is preferentially corroded, which is like NiFe_2_O_4_-10NiO ceramic and 15(Cu-20Ni)/NiFe_2_O_4_-10NiO cermet anode [4]. The decrease of Fe element content of the uncorroded NiFe_2_O_4_ proves to be a reaction between Fe element and NiO [20].

**Figure 8:**
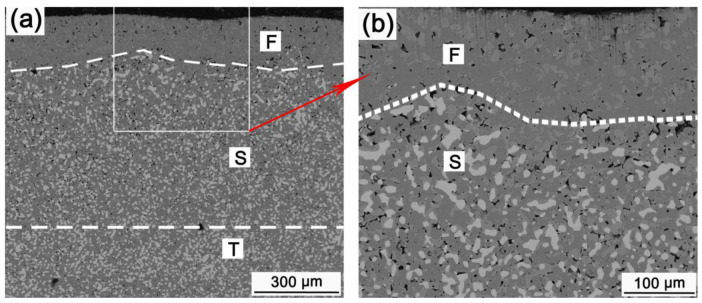
(**a**) Micrograph of HT-48-25, (**b**) is the magnification of selection zone in (**a**).

Figure 8 shows the bottom microstructure of HT-48-25. It also has three different morphology zones, i.e., F, S, and T. Porous zone P shown in Figure 6 is not found in the surface of the anode. The corrosion depth including internal oxidation corrosion part (F layer and S layer) is about 700 μm. Compared with that of HT-48-15, the corrosion depth increased by approximately 170 μm. Zone F without metallic phase is dense and approximately 170 μm thick. The NiO phase is much and coarse, similar to zone D in Figure 6. Although some pores are still found in zone S, the size of the pores is much smaller than that of zone P, shown in Figure 6. Moreover, no electrolyte is found in the pores.

From Figure 6 and Figure 8, it is clear that a dense ceramic layer can be formed at the bottom of the anode electrolysed at 960 °C, and the dense ceramic layer thickens as the electrolysis process proceeding. In general, the higher the temperature, the greater the physical dissolution and corrosion rate of ceramic phase, and the more difficult it is to form a dense layer. However, a dense ceramic surface can be formed at 960 °C but not at 880 °C, indicating that the effect of electrolyte composition on the formation of the dense ceramic layer is much more important than that of the temperature. Reducing the electrolysis temperature through modifying the electrolyte composition can alleviate the physical dissolution rate of the ceramic phase. However, the electrolysis temperature is primarily reduced by increasing the AlF_3_ concentration in the electrolyte, and a high AlF_3_ concentration will aggravate the chemical corrosion of the ceramic phase [26]. When chemical corrosion is more severe than physical dissolution corrosion, the corrosion rate of the anode at low temperature electrolysis will be more severe than that at high temperature electrolysis. Additionally, the weight gain of NiFe_2_O_4_-based cermets oxidized in air follows a parabolic law [27]. The internal oxidation depth of PO-48 is about 45 μm. The dense oxide surface layer reaches 170 μm after 25 h electrolytic corrosion, which indicated that the oxidation under electrolytic conditions is more serious than that in air. Previous studies have confirmed that the dense ceramic surface is mainly caused by the oxidation of the metallic phase, the reaction of NiO phase with the Fe-rich NiFe_2_O_4_ phase, and the reaction of the aluminum dissolved in the molten salt with the ceramic phase, as shown in Formulations (4)–(6) [22,28]. The higher the temperature is, the easier these reactions are. Therefore, the dense ceramic surface layer is much easier to form in the 960 °C electrolysis process than that conducted at 880 °C.

2Ni + O_2_ = 2NiO (4)

(5) $${\mathrm{Ni}}_{1-x}{\mathrm{Fe}}_{x}O+\frac{x}{4}O_{2}=\frac{x}{2}{\mathrm{NiFe}}_{2}O_{4}+\left(1\;-\;\frac{3x}{2}\right)\mathrm{NiO}$$

(6) $${\mathrm{Ni}}_{1-y}{\mathrm{Fe}}_{y}{\mathrm{Fe}}_{2}O_{4}+\frac{3y}{2}\mathrm{NiO}+\frac{y}{4}O_{2}=\left(1+\frac{y}{2}\right){\mathrm{NiFe}}_{2}O_{4}$$

From the microstructure of the anode electrolyzed at 960 °C, the newborn aluminate is mainly distributed in the porous edge and zone D near the porous zone. The NiO phase in zone D is obviously more than that of the core. Therefore, the densification of the D layer is not caused by aluminate, but mainly caused by the oxidation of Ni, which is different from those anodes with low Ni content and low metallic phase content [22,28]. This shows that part of the NiO phase in Figure 7 contains NiFe_2_O_4_ precipitates, but some do not. NiO phase can dissolve a certain amount of Fe^2+^ at high temperature [5], and the anode reaction releases oxygen (atomic oxygen) during electrolysis, which can promote the internal oxidation and precipitation of solid-dissolved Fe^2+^ in the sintered NiO. NiO containing NiFe_2_O_4_ precipitates was formed during the preparation process, and the NiO without NiFe_2_O_4_ precipitates should be formed by the oxidation of the Ni element in the metallic phase during the electrolysis process. The Fe^2+^ ion concentration in NiO formed by oxidation is much smaller than that in the sintered state, so there is no obvious NiFe_2_O_4_ precipitate inside the NiO particles generated by the oxidation. The oxidation product NiO nucleates and grows on the added NiO, so the NiO in zone D is larger than the as-sintered NiO. Since there is no electrolyte in zone D and the Ni/Cu atom ratio decreases, the Ni element should not undergo electrochemical preferential corrosion, but in-situ oxidation.

Table 3 shows that the Fe/Ni atomic ratio in NiFe_2_O_4_ phase in zone D is lower than that of the core zone, which is closer to the theoretical value of 2. Preferential corrosion of Fe is occurring at the begin of NiFe_2_O_4_ phase corrosion. As the Fe content decreases, the thermal stability of NiFe_2_O_4_ phase reduces, and then NiO, the fine precipitation phase as shown in Figure 6e precipitates. A fragmented morphology of NiFe_2_O_4_ phase is established when the NiO is corroded.

Overall, the corrosion process of the anode at 960 °C is as follows: First, Cu is externally oxidized, and Ni is internally oxidized. Then, metal oxides corrode, and Cu oxides preferentially dissolve and corrode. Part of NiO transforms into NiFe_2_O_4_ spinel, part of NiO reacts with the electrolyte to form NiAl_2_O_4_ spinel. On the one hand, Fe in NiFe_2_O_4_ phase reacts with NiO, and on the other hand, Fe element is preferentially corroded, which leads to a decrease in the stability of NiFe_2_O_4_ and the precipitation of NiO phase from NiFe_2_O_4_ phase. As the NiO phase gradually corrodes, NiFe_2_O_4_ phase collapse and is corroded. Because the oxidation of the anode is faster than the dissolution corrosion and chemical corrosion of the ceramic phase, and the internal oxidation of the metallic phase can make up the pores formed by the external diffusion oxidation and the electrolyte penetration channels, a dense metal-free ceramic layer could be formed.

## 4. Conclusions

Here, 20 Cu-20 Ni-54 NiFe_2_O_4_-6 NiO cermets anodes treated with different pre-oxidation time were electrolyzed at 880 and 960 °C for different times, and the corrosion behavior was studied. The following conclusions are obtained:

(1) The ceramic phase undergoes serious chemical corrosion when electrolyzed at 880 °C. Extending the pre-oxidation time can delay the rapid electrochemical corrosion of the anode. However, since the corrosion of the ceramic phase is faster than the oxidation of the metallic phase, the oxide scale is gradually dissolved, and it is difficult to form a dense ceramic surface layer.
(2) A dense ceramic surface layer can be formed on the bottom of the anode electrolyzed at 960 °C, and the dense layer thickens with the extension of the electrolysis time. The formation of the dense surface layer is mainly caused by the oxidation of Ni.
(3) The preferential corrosion of Fe element occurs first in the corrosion process of NiFe_2_O_4_ phase, and then the NiO phase is precipitated from NiFe_2_O_4_ phase. After the NiO is dissolved and corroded, the NiFe_2_O_4_ grains collapse and the ceramic phase peels off from the anode.

## Figures and Tables

**Table 1:** Composition and parameter of the electrolyte used in electrolysis test.

Electrolysis Temperature/°C	Electrolyte Composition/wt%						Melting Point/°C	Solubility of Al_2_O_3_/wt%
	Na_3_AlF_6_	K_3_AlF_6_	AlF_3_	Al_2_O_3_	CaF_2_	LiF		
880	51.39	17.13	24.0	4.48	/	3	875	4.23
960	78.07	/	9.5	7.43	5.0	/	947	7.13

**Table 2:** Anodes used for the electrolysis test.

Anode	Pre-Oxidation Time, h	Electrolysis Time, h	Electrolysis Temperature, °C
LT-0-15	0	15	880
LT-4-15	4	15	880
LT-4-25	4	25	880
LT-24-25	24	25	880
HT-48-15	48	15	960
HT-48-25	48	25	960

**Table 3:** Fe/Ni atomic ratios of NiFe_2_O_4_ and NiO phases at different locations in Figure 6.

Position	NiFe_2_O_4_	NiO
Zone P in Figure 6b	1.94	0.08
Zone D in Figure 6b	2.07	0.16
Zone S in Figure 6a	2.48	0.15
Zone T in Figure 6a	2.89	0.2

## Data Availability

All data included in this study are available upon request by contact with the corresponding author.
